# Severe Hypoxic and Hypercapnic Respiratory Failure Complicated by Mycobacterium Chimaera Intracellulare in a Patient with Gastric Pull-Up and COPD

**DOI:** 10.51894/001c.144429

**Published:** 2025-09-30

**Authors:** Mubera Bebanic

**Affiliations:** 1 Department of Internal Medicine Detroit Medical Center-Sinai Grace Hospital

## 68

### INTRODUCTION

Non-tuberculous mycobacteria (NTM), including Mycobacterium chimaera intracellulare, are rare but significant pathogens that can worsen respiratory outcomes in patients with chronic medical conditions. We present a case of recurrent respiratory failure in a patient with a history of gastric pull-up surgery and COPD, ultimately diagnosed with active Mycobacterium chimaera intracellulare infection.

### CASE DESCRIPTION

A 55-year-old male with advanced COPD on 4 L nasal cannula at home and a history of gastric pull-up for childhood caustic esophageal injury presented with dyspnea, hypoxia, and hypercapnia. On admission, he was tachycardic (HR 123 bpm), tachypneic (RR 22), and hypoxic (SpO₂ 89% on 6 L NC), with ABG showing pH 7.27, pCO₂ 126 mmHg, and pO₂ 63 mmHg on BiPAP. Physical exam revealed cachexia, diminished breath sounds bilaterally (left greater than right), and bilateral pitting edema. Chest X-ray showed extensive left lower lobe pneumonia, bilateral pleural effusions, and a chronic right upper lobe cavitary lesion, confirmed by CT as slightly enlarged compared to imaging in 2020. Labs showed WBC 11.6 k/μL, hemoglobin 10.3 g/dL, and lactic acid 2.5 mmol/L. He had a similar presentation in 2020 with multiple cavitary lesions, where cultures grew Mycobacterium chimaera intracellulare. Empiric vancomycin and Zosyn were initiated but later transitioned to Unasyn per infectious disease recommendations. Repeat sputum cultures confirmed active NTM infection. Rifampin, ethambutol, and azithromycin were recommended for 12-18 months. This case highlights the need for comprehensive follow-up and multidisciplinary management in patients with recurrent respiratory failure and structural lung diseases. His gastric pull-up history predisposed him to aspiration pneumonia and severe bullous emphysema, complicating his course. Reactivation of Mycobacterium chimaera intracellulare underscores the importance of timely recognition and prolonged treatment of NTM infections, even in immunocompetent patients.

### CONCLUSION

This case illustrates the interplay of COPD, anatomical alterations, and NTM infections in recurrent respiratory failure. It underscores the need to recognize Mycobacterium chimaera intracellulare in patients with cavitary lung lesions and highlights the role of infectious disease consultation in guiding appropriate therapy.

